# STD Awareness Month — April 2013

**Published:** 2013-04-05

April is STD Awareness Month, an annual event calling attention to the impact of sexually transmitted diseases (STDs) in the United States. This month-long observance provides individuals, doctors, and community-based organizations an opportunity to address ways to prevent some of nearly 20 million new cases of STDs that occur in the United States each year (1), costing the U.S. health-care system nearly $16 billion in direct medical costs (2), and placing a substantial human and economic burden on the nation.

STDs can lead to serious health problems if not diagnosed and treated early. Undetected and untreated STDs can increase a person’s risk for human immunodeficiency virus infection and cause other serious health consequences, such as infertility. However, most STDs are treatable, and many are curable. STD screening can help detect disease early and, when combined with appropriate treatment, is one of the most effective tools available to protect one’s health and prevent the spread of STDs to others.

Additional information about STDs and resource materials that can be shared with patients and community members are available at http://www.cdc.gov/std.
